# Long non-coding RNA *MIR4435-2HG*: a key molecule in progression of cancer and non-cancerous disorders

**DOI:** 10.1186/s12935-022-02633-8

**Published:** 2022-06-17

**Authors:** Majid Ghasemian, Masoumeh Rajabibazl, Unes Sahebi, Samira Sadeghi, Reza Maleki, Veys Hashemnia, Reza Mirfakhraie

**Affiliations:** 1 Department of Clinical Biochemistry, Faculty of Medicine, Shahid Beheshti University of Medical Sciences, Tehran, Iran; 2 Department of Biology, Faculty of Science, University of Guilan, Rasht, Iran; 3 Department of Medical Genetics, Faculty of Medicine, Shahid Beheshti University of Medical Sciences, Tehran, Iran; 4 Hematopoietic Stem Cell Research Center, Shahid Beheshti University of Medical Sciences, Tehran, Iran

**Keywords:** *MIR4435-2HG*, lncRNA, Cancer, Biomarker

## Abstract

*MIR4435-2HG* (*LINC00978*) is a long non-coding RNA (lncRNA) that acts as an oncogene in almost all cancers. This lncRNA participates in the molecular cascades involved in other disorders such as coronary artery diseases, osteonecrosis, osteoarthritis, osteoporosis, and periodontitis. *MIR4435-2HG* exerts its functions via the spectrum of different mechanisms, including inhibition of apoptosis, sponging microRNAs (miRNAs), promoting cell proliferation, increasing cell invasion and migration, and enhancing epithelial to mesenchymal transition (EMT). *MIR4435-2HG* can regulate several signaling pathways, including Wnt, TGF-β/SMAD, Nrf2/HO-1, PI3K/AKT, MAPK/ERK, and FAK/AKT/β‑catenin signaling pathways; therefore, it can lead to tumor progression. In the present review, we aimed to discuss the potential roles of lncRNA *MIR4435-2HG* in developing cancerous and non-cancerous conditions. Due to its pivotal role in different disorders, this lncRNA can serve as a potential biomarker in future investigations. Moreover, it may serve as a potential therapeutic target for the treatment of various diseases.

## Introduction

Genetic alterations are one of the primary causes of cancer, leading to the deregulation of gene networks [1–3]. In recent years following developments in RNA sequencing technologies, this insight came into being that a large part of the genome transcribes into non-protein-coding RNAs [4]. Long non-coding RNAs (lncRNAs) are a subclass of functional RNAs which are longer than 200 nucleotides in sequence length without a protein-coding capacity [5–7]. In the beginning, lncRNA transcripts were regarded as ‘transcriptional noise’ or ‘junk’. Subsequent investigations revealed that lncRNAs are key players in human disorders, particularly in malignant conditions [8, 9]. Although lncRNAs do not translate into proteins, they play a meaningful function in regulating gene expression through different mechanisms such as remodeling of chromatin, modulating the activity of transcription factors, epigenetic regulation, post-transcriptional, and cell cycle regulation [10, 11]. *MIR4435-2 Host Gene (MIR4435-2HG)*, also named *LINC00978*, *AK001796*, *AWPPH*, *MIR4435-1HG*, *MORRBID*, and *AGD2*, is an lncRNA that resides on chromosome 2q13 region and includes ten exons. *MIR4435-2HG* has 108 transcripts produced through alternative splicing (https://www.ensembl.org/Homo_sapiens/Gene/Summary?db=core;g=ENSG00000172965;r=2:111006015-111523376). Previous studies have reported that the *MIR4435-2HG* has an oncogenic role in the progression of different cancer types. In addition to the role of *MIR4435-2HG* in tumorigenesis, some studies suggest that it is involved in the pathogenesis of non-cancerous conditions such as coronary artery diseases [12], osteonecrosis [13], osteoarthritis [14], osteoporosis [15], and periodontitis [16]. Due to the important regulatory roles of *MIR4435-2HG*, in the present review, we provide comprehensive information about its function in cancer and other diseases.

## *MIR4435-2HG* and cancer

Previous studies have shown that the expression level of *MIR4435-2HG* was upregulated in almost all cancers. *MIR4435-2HG* upregulation can promote tumor progression by increasing cell proliferation**,** invasion, migration, epithelial-mesenchymal transition (EMT), chemoresistance and suppression of apoptosis.

### Colorectal cancer (CRC)

**Fig. 1:**
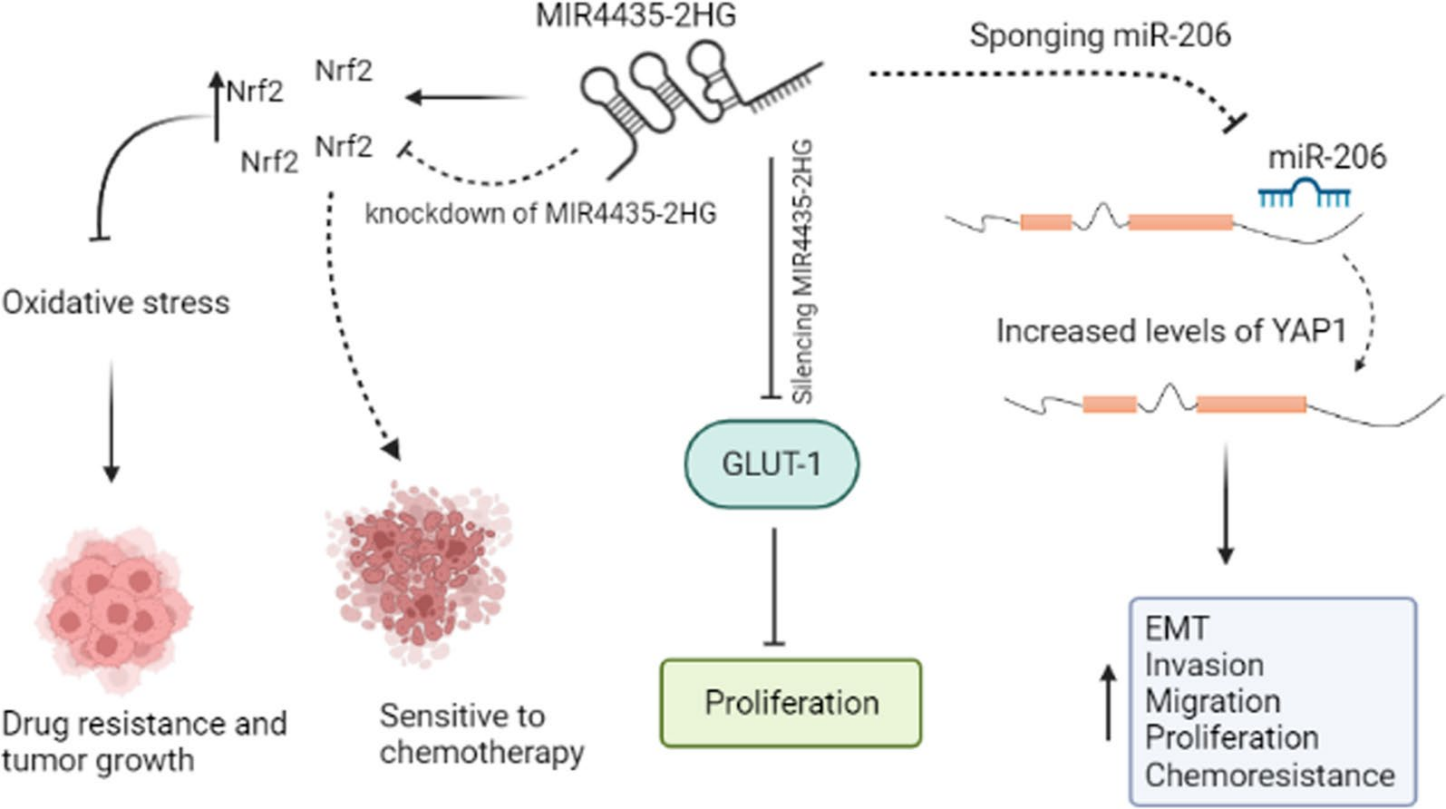
Schematic representation of *MIR4435-2HG* functions in CRC. Increased level of *MIR4435-2HG* blocks *miR-206* which leads to elevation of *YAP1* and enhanced cell invasion, migration, proliferation and chemoresistance. In HCT116R cells, the activation of Nrf2 pathway leads to drug resistance; however, knockdown of *MIR4435-2HG* sensetives cancer cell to chemotherapy through inhibition of Nrf2. Furthermore, *MIR4435-2HG* can increase CRC progression via promoted *GLUT-1*

Overexpression of *MIR4435-2HG* has been reported in CRC tissues and cell lines in several studies [17–22]. Wen et al*.* have demonstrated that upregulation of *MIR4435-2HG* in CRC tissues was significantly correlated with the TNM stage [17]. Cancer-developing conditions such as chemoresistance, invasion, metastasis, migration, cancer stemness, and EMT can be regulated by Yes-related protein 1 (YAP1) transcription factor [23]. Dong et al*.* showed that *MIR4435-2HG* could regulate the expression of *miR-206*. On the other hand, they also indicated that *YAP1* was a potential target for *miR-206* (Fig. 1 and Table 1). *MIR4435-2HG* knockdown could block invasion, migration, and cell proliferation through the miR-206/YAP1 axis in the HCT116 and SW620 cell lines [18]. Previous studies reported that expression of nuclear factor erythroid 2-related factor 2 (Nrf2) and its regulator, heme oxygenase-1 (HO-1), increased after treating cancer cells with chemotherapeutic agents. These factors regulate the detoxification process and antioxidant enzymes, which results in the reduction of drug effects and an increase in drug resistance [24, 25]. In HCT116R cells (a cisplatin-resistant cell line), knockdown of *MIR4435-2HG* significantly induced cisplatin sensitivity, enhanced apoptosis, and inhibited cell proliferation via modulating Nrf2/HO-1 cascade (Fig. 1). Hence, it seems that *MIR4435-2HG* is involved in oxidative stress [19]. Another experiment has indicated that in patients with colon cancer, serum levels of *glucose transporter 1* (*GLUT‑1)* and *MIR4435-2HG* were significantly higher than healthy controls. Moreover, silencing *MIR4435-2HG* inhibits cell proliferation through downregulation of *GLUT-1* in the HT-29 cancerous cell line (Fig. 1) [20]. In our previous study, we showed a positive correlation between *β-catenin* and *MIR4435-2HG* expression that indicated mentioned lncRNA might regulate the Wnt signaling pathway via stabilization of *β-catenin,* which can lead to the progression of CRC [21]. *Shen *et al*.* showed that high expression of *MIR4435-2HG* was remarkably related to clinicopathological features, including stage, tumor size, tumor node and lymph node metastasis. Their results showed that the patients with higher levels of *MIR4435-2HG* had a worse prognosis than the patients with lower expression levels. In addition, *MIR4435-2HG* silencing remarkably reduced cell proliferation and enhanced cell apoptosis [22]. To conclude, *MIR4435-2HG* can promote CRC via different mechanisms.

**Table 1:** *MIR4435-2HG* participates in the pathogenesis of different cancers via the regulation of different miRNAs (∆: knock-down, EMT: Epithelial-Mesenchymal Transition, TNBC: Triple‐negative breast cancer, NSCLC: non‑small cell lung cancer, HNSCC: head and neck squamous cell carcinoma)

Cancer type	MIR4435-2HG	miRNA	Target gene	Function	References
Colorectal cancer	Up-regulated	↓miR-206	↑YAP1	∆ MIR4435-2HG: ↓Invasion, ↓Migration, ↓Cell proliferation, ↓EMT, ↓CRC growth, ↓Liver metastasis	[18]
Gastric cancer	Up-regulated	↓miR-497	↑ *NTRK3*	∆ MIR4435-2HG: ↓Cell proliferation, ↓Metastasis, ↑Apoptosis	[28]
Gastric cancer	Up-regulated	↓miR-138-5p	↑SOX4	∆ MIR4435-2HG: ↓Invasion, ↓Migration, ↓Cell proliferation, ↓EMT, ↑Apoptosis, ↓Tumor growth	[29]
Hepatocellular carcinoma	Up-regulated	↑*miRNA-487a*	–	↑MIR4435-2HG: ↑*miRNA-487a,* ↑Cell proliferation,	[35]
Hepatocellular carcinoma	Up-regulated	↓miR‑136‑5p	↑B3GNT5	∆ MIR4435-2HG: ↓Invasion, ↓Migration, ↓Cell proliferation	[38]
NSCLC	Up-regulated	↓miR-6754-5p	–	∆ MIR4435-2HG: ↓Invasion, ↓Migration, ↓Cell proliferation, ↑Apoptosis	[45]
Breast cancer	Up-regulated	↓miR-22-3p	↑TMEM9B	∆ MIR4435-2HG: ↓viability, ↓Invasion, ↓Migration, ↓Cell proliferation, ↓EMT	[48]
Ovarian cancer	Up-regulated	↓miR-128-3p	↑CDK14	∆ MIR4435-2HG: ↓Cell proliferation, ↓Invasion, ↓Migration, ↓Tumor growth, ↑Apoptosis	[51]
Glioma cancer	Up-regulated	↓miR-1224-5p	↑TGFBR2	∆ MIR4435-2HG: ↓Cell proliferation, ↓Invasion, ↓Tumor growth	[58]
Glioma cancer	Up-regulated	↓miR- 125a- 5p	↑TAZ	∆ MIR4435-2HG: ↓Migration, ↓Cell proliferation, ↑Apoptosis, ↓Wnt pathway, ↓Tumor volume	[61]
Cervical cancer	Up-regulated	↓miR-128-3p	↑MSI2	∆ MIR4435-2HG: ↓Cell proliferation, ↓Invasion, ↓Migration	[66]
Bladder cancer	Up-regulated	↓miR-4288	–	∆ MIR4435-2HG: ↓Cell proliferation, ↓Invasion, ↓Migration	[71]
HNSCC	Up-regulated	↓miR‑383‑5p	↑RBM3	∆ MIR4435-2HG: ↓Cell proliferation, ↓Invasion, ↓Migration, ↓EMT, ↓Tumor growth	[70]
Melanoma	Up-regulated	↓miR-802	↑FLOT2	↑MIR4435-2HG: ↑Cell proliferation, ↑Invasion, ↑Migration	[72]
TNBC	Up-regulated	↑miRNA‐21	-	↑MIR4435-2HG: ↑cell viability, ↑cell proliferation, ↑chemoresistance	[49]
NSCLC	Up-regulated	↓miRNA-204	↑CDK6	∆ MIR4435-2HG: ↓cell proliferation, ↓invasion, ↓migration	[46]

### Gastric cancer (GC)

**Fig. 2:**
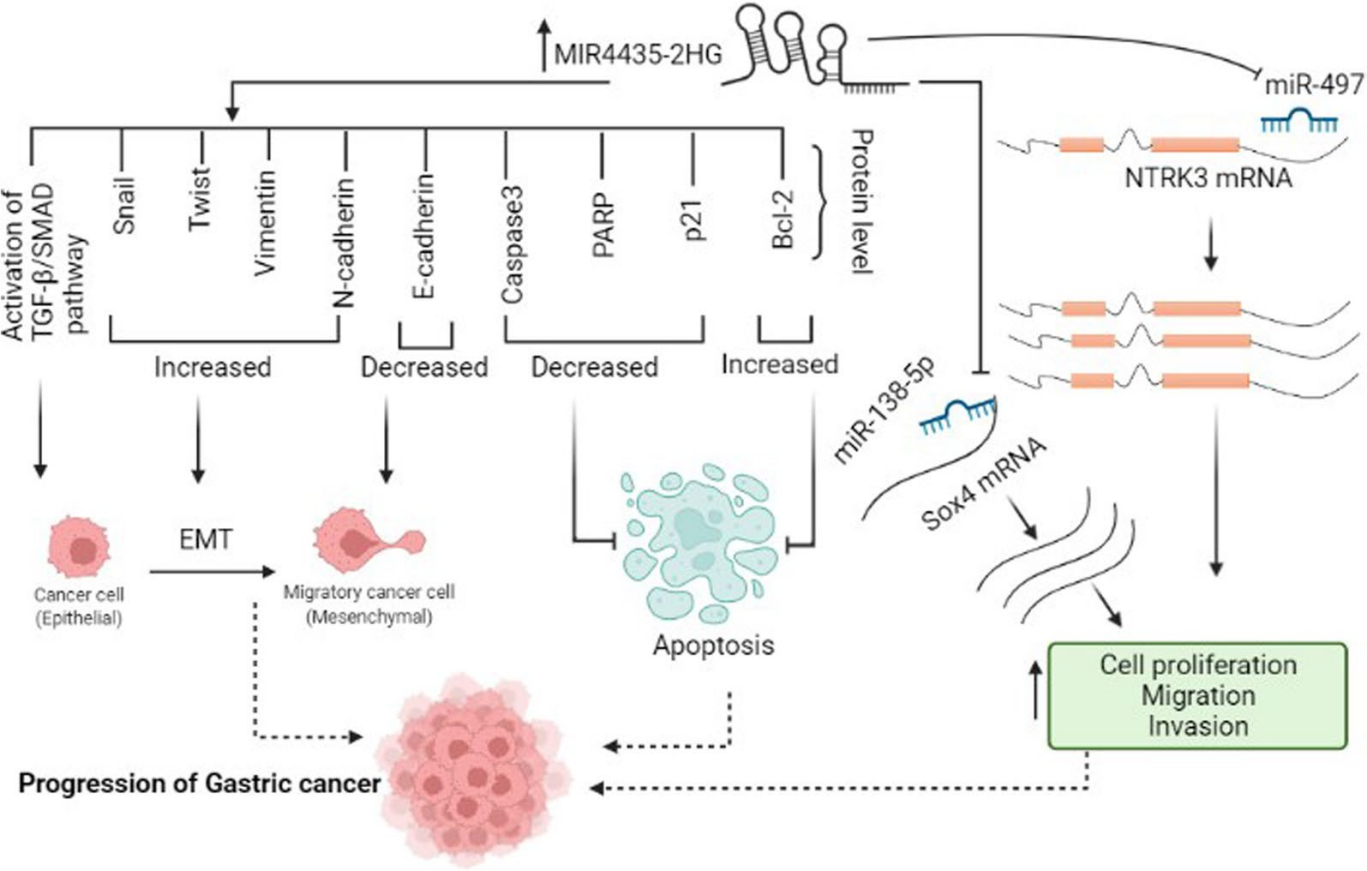
*MIR4435-2HG* exerts its oncogenic role via different mechanisms in the progression of GC. *MIR4435-2HG* acts as a molecular sponge for miR-497 and miR-138-5p; therefore, it enhances the expression of *NTRK3* and *SOX4* (respectively), which results in cell proliferation, migration, and invasion. Also, an elevated level of *MIR4435-2HG* activates the TGF-β/SMAD signaling pathway, promotes EMT and suppresses apoptosis

Several studies reported that the expression level of *MIR4435-2HG* was significantly increased in GC tissues, plasma samples, and different cell lines compared to the normal controls [26–29]. TGF-β/SMAD is one of the pathways involved in the progression of metastasis in gastric cancer [30]. Min et al*.* showed that *MIR4435-2HG* expression was significantly correlated with TNM stage, tumor size, and lymphatic metastasis. Knockdown of *MIR4435-2HG* elevated the expression of E-cadherin protein while the expression levels of vimentin, slug, N-cadherin, and twist proteins were inhibited. On the other hand, *MIR4435-2HG* knockdown leads to the inhibition of transforming growth factor beta (TGF-β) and phosphorylated SMAD2 (p-SMAD2) in gastric cancer cell lines. This observation suggests that knockdown of *MIR4435-2HG* can elevate EMT, and apoptosis and inhibit cell cycle progression, invasion, and migration via the regulation of TGF-β/SMAD signaling pathway (Fig. 2) [27, 31, 32]. Yuan et al*.* reported that *MIR4435-2HG* could target *miR-497*. Interestingly, tropomyosin receptor kinase C (*NTRK3*) plays a critical role in cancer progression and is a direct target of *miR-497*. *MIR4435-2HG* acts as a molecular sponge of *miR-497*, which leads to an increase in *NTRK3*. It can be concluded that the elevation of *MIR4435-2HG* could enhance tumorigenesis via miR-497/NTRK3 axis (Fig. 2 and Table 1) [28]. Gao et al*.* demonstrated that high expression of *MIR4435-2HG* was associated with poor survival rate in GC patients. They also reported the enhancement of apoptosis and suppression of cell proliferation, migration, invasion and EMT after *MIR4435-2HG* knockout in gastric carcinoma cells. It was suggested that overexpression of *MIR4435-2HG* affects the expression of *SRY-box transcription factor 4(SOX4)* via sponging miR-138-5p. Therefore, *MIR4435-2HG* plays an oncogenic role in GC by targeting the miR-138-5p/SOX4 axis (Fig. 2 and Table 1) [29].

### Hepatocellular carcinoma (HCC)

**Fig. 3:**
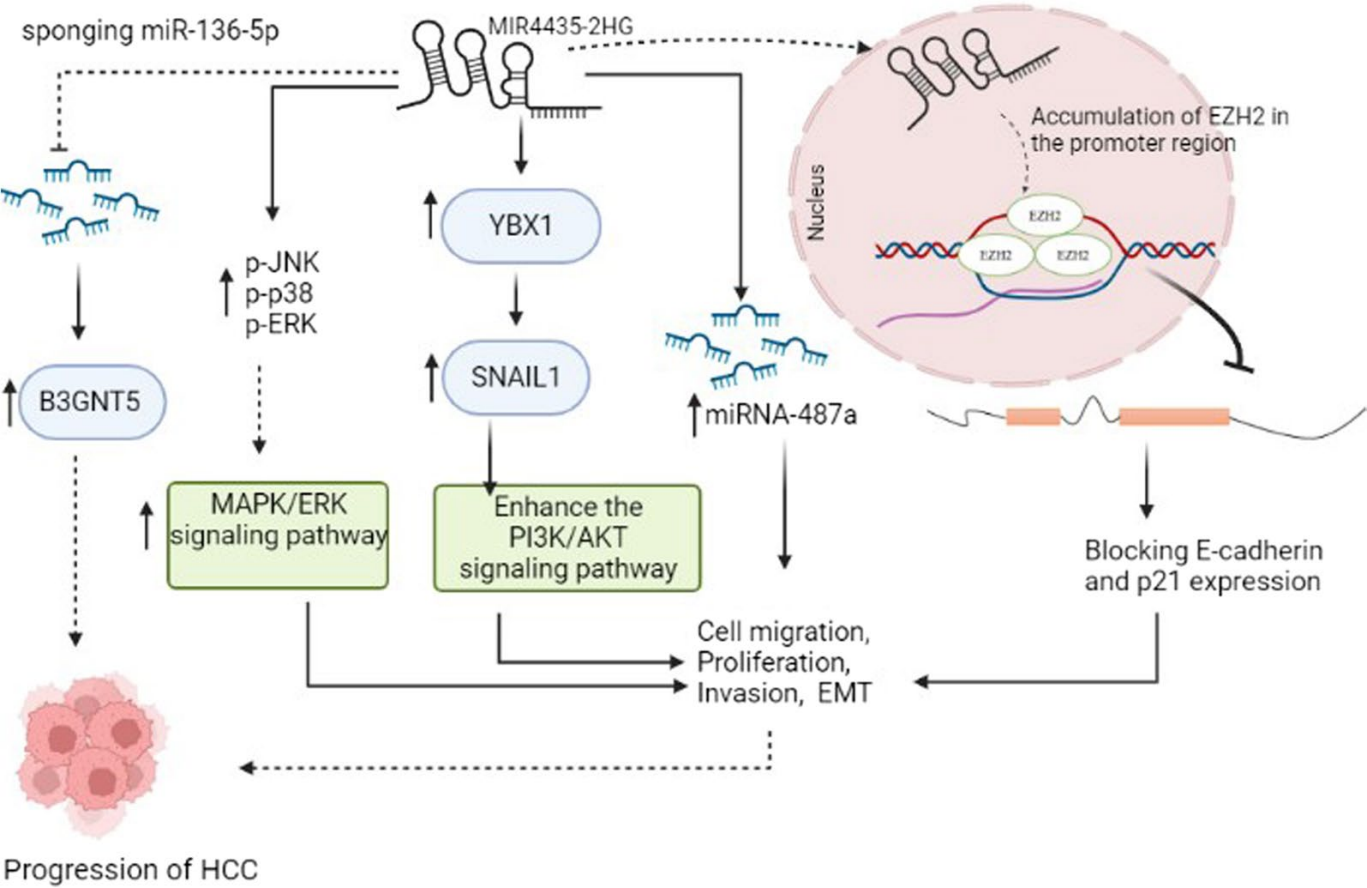
The impact of *MIR4435-2HG* in the progression of HCC through different mechanisms. *MIR4435-2HG* increases promoter methylation of *E-cadherin* and *p21* via mediating the accumulation of EZH2 in the promoter region. *MIR4435-2HG* increases HCC progression via blocking *E-cadherin* and *p21* expression. In addition, *MIR4435-2HG* promotes cell proliferation, migration, invasion, and EMT via activation of MAPK/ERK and PI3K/AKT signaling pathways. The effect of *MIR4435-2HG* is exerted via sponging miR-136-5p and interaction with miR-487a

*MIR4435-2HG* upregulation has also been detected in hepatocellular carcinoma tissues and cell lines. [33–38]. I*n vitro* and in vivo studies performed by Zhao et al*.* showed that upregulation of *MIR4435-2HG* increased migration, cell proliferation, metastasis, and tumor growth in hepatocellular carcinoma cells via regulating the interaction of Y-box binding protein 1 (YBX1) with snail family transcriptional repressor 1 (SNAIL1) and phosphatidylinositol-4,5-bisphosphate 3-kinase catalytic subunit alpha)PIK3CA(. Previous studies reported that YBX1 could induce EMT, SNAIL1 mRNA translation, and promote metastasis. YBX1 can stimulate PIK3CA transcription and enhance the PI3K/AKT signaling pathway by binding its promoter in cancer cells (Fig. 3) [34, 39, 40]. Another study showed that *miRNA-487a* and *MIR4435-2HG* were elevated in HCC tumor samples compared to adjacent tissues, and a positive correlation was detected between the genes expression. The overexpression of *MIR4435-2HG* in the HCC SNU-398 and SNU-182 cell lines promoted cell proliferation through upregulation of *miRNA-487a* (Fig. 3 and Table 1) [35]. Polycomb repressive complex 2 (PRC2) consists of multiple subunits including, Enhancer of Zeste Homolog 2 (EZH2) that displays methyltransferase activity. Previous studies showed that EZH2 was remarkably upregulated in many cancers, including HCC*.* Using chromatin immunoprecipitation (ChIP), Xueying et al*.* showed that *E-cadherin* and *p21* are molecular targets of *MIR4435-2HG*. As shown in Fig. 3, *MIR4435-2HG* enhances the promoter methylation of *E-cadherin* and *p21* genes via mediating the accumulation of EZH2 in the promoter region. It can be concluded that *MIR4435-2HG* increases HCC progression via blocking *E-cadherin* and *p21* expression through EZH2-mediated epigenetic silencing (Fig. 3) [36, 41, 42]. Zhang et al*.* reported that high expression of *MIR4435-2HG* correlates with poor HCC prognosis. They also indicated that *MIR4435-2HG* knockdown strongly induced apoptosis, cell cycle arrest and significantly decreased HCC cell proliferation capacity. Inhibition of *MIR4435-2HG* led to a decrease of phosphorylated JNK (p-JNK), phospho-p38 (p-p38), and phospho-ERK (p-ERK). It seems that *MIR4435-2HG* induces the progression of HCC by activating the MAPK/ERK signaling pathway (Fig. 3) [37]. Zhu et al*.* identified the target genes of *MIR4435-2HG.* They also confirmed interactions between *MIR4435‑2HG*, *miR‑136‑5p*, and *B3GNT5*, one of the downstream targets of *miR‑136‑5p*, using luciferase reporter assays. *MiR‑136‑5p* acts as a tumor suppressor in various cancers such as liver cancer. *MIR4435‑2HG* could sponge *miR‑136‑5p* while the expression of *UDP-GlcNAc:betaGal beta-1,3-N-acetylglucosaminyltransferase 5 (B3GNT5)* was upregulated in liver cancer tissues. It can be concluded that *MIR4435‑2HG*, by sponging *miR‑136‑5p,* can directly reverse its inhibitory effects on target genes such as *B3GNT5*, thereby facilitates the progression of liver cancer via the *MIR4435‑2HG/miR‑136‑5p/ B3GNT5* axis (Fig. 3 and Table 1) [38].

### Lung cancer (LC)

Qiaoyuan et al*.* showed that the *MIR4435-2HG* expression was downregulated after treating LC cells with resveratrol. They showed that cell cycle arrest occurred in G0/G1 phase following *MIR4435-2HG* knockdown. They also indicated that inhibition of *MIR4435-2HG* in lung cancerous cell lines enhanced the anticancer effects of resveratrol [43]. Another experiment revealed EMT suppression following *MIR4435-2HG* knockdown. Notably, *MIR4435-2HG* prevents the destruction of β-catenin by the proteasome system, however, *MIR4435-2HG* knockdown resulted in the decreased β-catenin transactivation and subsequent inhibition of the Wnt/β-catenin signaling pathway [44]. *MIR4435-2HG* can potentially sponge *miR-6754-5p* in non‑small cell lung cancer (NSCLC). In NSCLC samples, the *miR-6754-5p* expression was downregulated and negatively correlated with *MIR4435-2HG* expression. It can be concluded that the *MIR4435-2HG* plays an oncogenic role in NSCLC via blocking the *miR-6754-5p* function (Table 1) [45]. Recently, Wu et al. introduced *miR-204* as a target for *MIR4435-2HG* in NSCLC. *MIR4435-2HG* leads to the progression of NSCLC through sponging *miR-204*. Silencing of *MIR4435-2HG* promoted the expression of *miR-204* and therefore decreased the expression of *cyclin dependent kinase 6 (CDK6),* resulting in the enhancement of cell proliferation, invasion and migration in the A549 cell line [46].

### Breast cancer (BC)

**Fig. 4:**
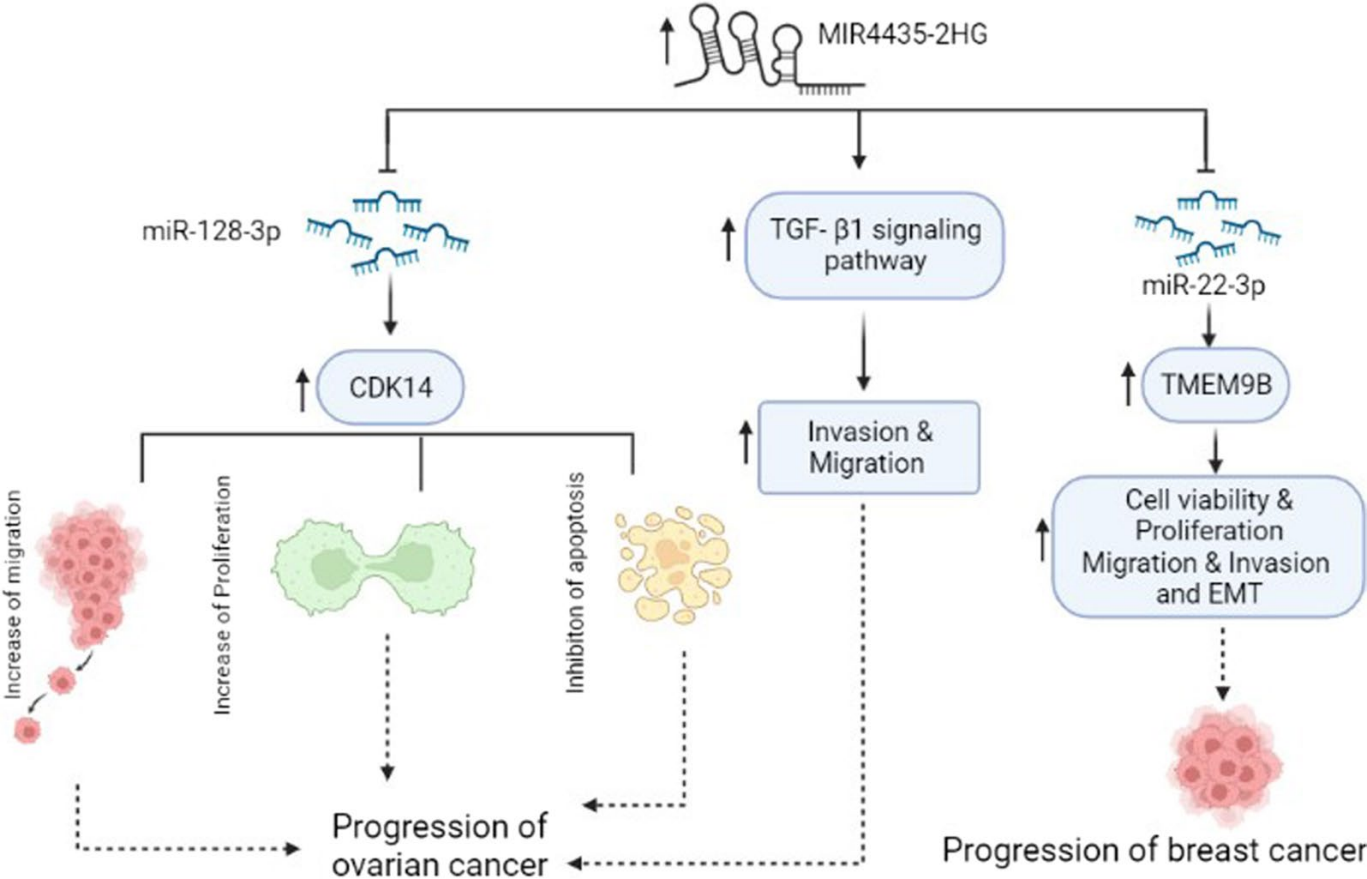
The role of *MIR4435-2HG* in the pathogenesis of ovarian and breast cancers. In ovarian cancer, *MIR4435-2HG* regulates migration, invasion and apoptosis through modulating the expression of *CDK14*. The effect of *MIR4435-2HG* is exerted via sponging miR-128-3p. In addition, *MIR4435-2HG* enhances the progression of ovarian cancer via activation of the TGF-β signaling pathway. In breast cancer, *MIR4435-2HG* sponges miR-22-3p and results in a subsequent increase in TMEM9B expression and tumorigenesis

One of the pioneer investigations for the assessment of *MIR4435-2HG* has been conducted in the BC tissues and cell lines by Lin et al. They indicated that *MIR4435-2HG* was over-expressed in breast cancer tissues and cell lines compared with corresponding controls and therefore may act as an oncogene. They reported that hormone receptor status and *MIR4435-2HG* expression were negatively correlated [47]. Consistent with the above study, Jing et al*.* showed that *MIR4435-2HG* was upregulated in breast cancer tissues and cell lines. They indicated that *MIR4435-2HG* could enhance many cellular parameters such as proliferation, EMT, migration, and invasion via regulating the miR-22-3p/TMEM9B axis (Fig. 4 and Table 1) [48]. Liu et al. demonstrated that the plasma level of *MIR4435-2HG* was remarkably higher in patients with Triple-negative breast cancer (TNBC) than healthy controls, and its expression level was positively correlated to *miR-21*. It was concluded that overexpression of *MIR4435-2HG* increased cell viability, proliferation and induced chemoresistance via interaction with *miR-21* in MDA-MB-231 and BT-20 cell lines [49].

### Ovarian cancer (OC)

It has been shown that *MIR4435-2HG* was upregulated in OC tissues and cell lines [50, 51]. It is suggested that *MIR4435-2HG* can distinguish stage I and II OC patients from healthy controls. Gong et al. reported that the expression level of TGF-β1 was upregulated in OC tissues and positively correlated with *MIR4435-2HG* expression*.* Using in vitro studies, they indicated the overexpression of *MIR4435-2HG* in UWB1.289 and UWB1.289 + BRCA1 cells led to upregulation of TGF- β1. Taken together, *MIR4435-2HG* could increase OC progression through overexpression of TGF-β1 (Fig. 4) [50]. Lijuan et al. indicated that the *MIR4435-2HG* and *cyclin dependent kinase 14 (CDK14)* were upregulated while *miR-128-3p* was down-regulated in cell lines and OC tissue samples. On the other hand, the expression of *MIR4435-2HG* was negatively associated with *miR-128-3p* in OC tissue. They showed that *MIR4435-2HG* could target *miR-128-3p* therefore, it might be concluded that *MIR4435-2HG* acts as the *miR-128-3p* sponge. *CDK14* is a downstream target of *miR-128-3p*. In vitro studies confirmed that *miR-128-3p* targeted *CDK14* and suppressed its expression. Knockdown of *MIR4435-2HG* promoted the expression of *miR-128-3p*, which led to decreased *CDK14* expression (Fig. 4 and Table 1) [51].

### Prostate cancer (PC)

**Fig. 5:**
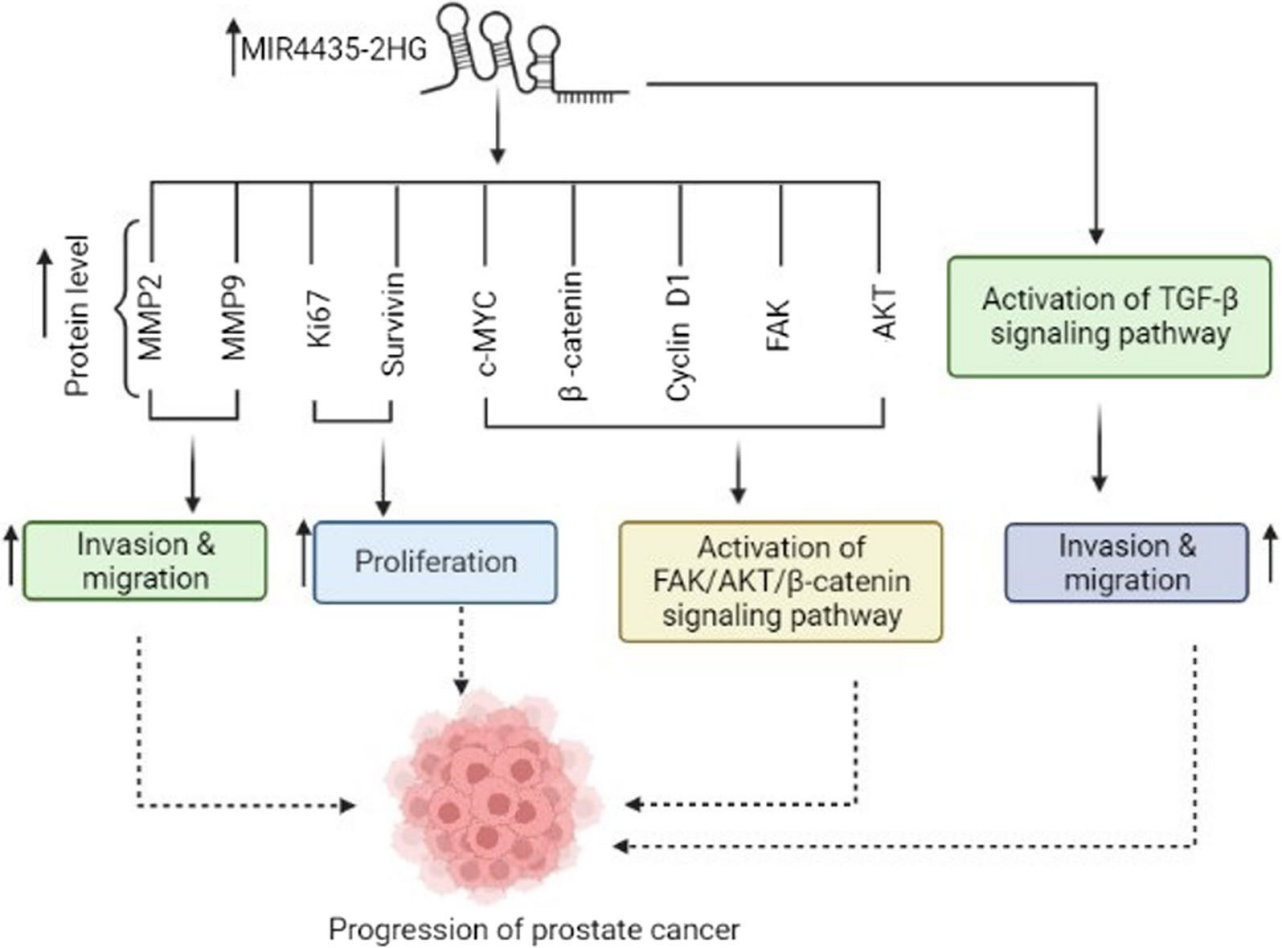
*MIR4435-2HG* contributes to the pathogenesis of prostate cancer via different mechanisms. Elevation of *MIR4435-2HG* induces cell proliferation, invasion, migration and activation of TGF-β and FAK/AKT/ β-catenin signaling pathways via modulating the expression of *MMP2*, *MMP9*, *Ki67*, *SURVIVIN*, *c-MYC*, *β-catenin*, *Cycline D1*, *FAK* and *AKT*

The expression level of *MIR4435-2HG* is reported to be enhanced in prostate cancer. It is suggested that this lncRNA causes cancer progression through various mechanisms such as FAK/AKT/β‑catenin and TGF-β1 pathways [52, 53]. Moreover, overexpression of *ST8 alpha-N-acetyl-neuraminide alpha-2,8-sialyltransferase 1* (*ST8SIA1)* can increase the tumor cell proliferation, migration, and invasion in prostate cancer, colorectal cancer, and breast cancer via the promotion of the FAK‑AKT‑mTOR signaling pathway [52, 54, 55]. Knockdown of *MIR4435-2HG* suppressed cell proliferation, invasion, and migration by blocking the activation of the FAK/AKT/β‑catenin pathway in PC cell lines. Xing et al. indicated that knockdown of *ST8SIA1* suppressed the effects of *MIR4435-2HG* in tumor progression. It seems that *MIR4435-2HG* contributes to tumorigenesis via the *MIR4435-2HG*/*ST8SIA1* axis [52, 56]. Hui et al. demonstrated that the plasma level of TGF- β1 was remarkably higher in patients with PC than healthy controls, and *TGF- β1* expression level was positively correlated to *MIR4435-2HG*. They reported that the effects of *MIR4435-2HG* on cell migration and invasion decreased following the inhibition of *TGF-β1* (Fig. 5) [53].

### Glioma cancer

One of the members of the TGF-β/Smad signaling pathway is transforming growth factor-beta receptor type II (TGFBR2) that acts as a cancer suppressor [57]. Xu et al. reported that the expression level of *MIR4435-2HG* was upregulated in patients with glioblastoma (GBM). In contrast, the expression level of *miR-1224-5p* was suppressed in GBM cancer cell lines. They used bioinformatics predictions and in vitro methods to show that this lncRNA acts as a sponge for *miR-1224-5p*. On the other hand, one of the direct targets of *miR-1224-5p* is *TGFBR2* gene, and the mRNA level of its gene was enhanced in GBM cancer cell lines. Taken together, it can be argued that *MIR4435-2HG* can promote cancer progression by targeting the *miR-1224-5p/TGFBR2* axis (Table 1) [58]. TGF-β signaling pathway is an essential factor for EMT. In patients with glioma cancer, a positive correlation was detected between the plasma levels of *TGF-β* and *MIR4435-2HG*. Therefore, it may be concluded that *MIR4435-2HG* is involved in the progression of glioma occur through TGF-β signaling pathway [59]. As a transcription coactivator, tafazzin, phospholipid-lysophospholipid transacylase (TAZ) is one of the most important downstream effectors of the Hippo signaling pathway that regulates cell proliferation, migration, and apoptosis [60]. The expression level of TAZ was upregulated in the brain tissue of glioma patients. Shen et al. indicated that the expression of *MIR4435- 2HG* was positively correlated with TAZ expression, while *miR- 125a- 5p* expression was negatively correlated with TAZ expression in the brain tissue (Table 1) [61].

### Leukemia cancers

Rho‑associated protein kinase 2 (ROCK2) is an important therapeutic target, and its upregulation was confirmed in many cancers, including T‑cell acute lymphoblastic leukemia (T‑ALL) [62, 63]. The expression of *MIR4435-2HG* and *ROCK2* was positively correlated in patients with T‑ALL. The overexpression of *MIR4435-2HG* remarkably increased *ROCK2* expression at both protein and mRNA levels; also, the overexpression of *ROCK2* significantly upregulated *MIR4435-2HG* expression in T‑ALL cells. It seems that *MIR4435-2HG* inhibits apoptosis and increases cell proliferation in T‑ALL cells through interactions with *ROCK2* [64]. Zhigang et al*.* reported that *MIR4435-2HG* was overexpressed in human acute myeloid leukemia (AML), which was correlated with a poor survival rate. They showed that *MIR4435-2HG* acts as a transcriptional repressor of *BIM* pro-apoptotic gene and, via this mechanism, regulates the lifespan of myeloid cells. In vivo study demonstrated that the loss of *MIR4435-2HG* in genetic mice models promoted the expression levels of *BIM* that increased cell death in mature and immature myeloid cells [65].

### Other cancers

The overexpression of *MIR4435-2HG* has also been reported in other cancers, including cervical cancer (CC) [66], clear cell renal cell carcinoma (ccRCC) [67, 68], esophageal squamous cell (ESCC) [69], head and neck squamous cell carcinoma (HNSCC) [70], bladder cancer (BCa) [71], melanoma [72] and nasopharyngeal carcinoma (NPC) [73].

*MIR4435-2HG* could target *miR-128-3p* and negatively modulated its expression in cervical cancer (CC). As shown in Fig. 5d, *miR-128-3p* negatively regulates the expression level of *musashi RNA binding protein 2 (MSI2)* gene. Therefore, it can be concluded that knockdown of *MIR4435-2HG* suppresses migration, invasion, and proliferation of CC cells via regulating the *miR-128-3p/MSI2* axis (Table 1) [66]. Jianquan et al*.* reported that *MIR4435-2HG* knockdown not only increased apoptosis and cell cycle arrest in G0/G1 phase but also decreased invasion and migration in clear cell renal cell carcinoma [67]. Zhu et al*.* suggested that *MIR4435-2HG* could directly interact with miR-513a-5p and repressed its expression in ccRCC. Knockdown of *MIR4435-2HG* inhibited proliferation, metastasis and tumour progression by downregulating *Kruppel like factor 6 (KLF6)* as the direct target of *miR-513a-5p* [68].

Knockdown of lncRNA *MIR4435-2HG* regulates cell cycle, cell proliferation, and cells growth via modulating MDM2/p53 signaling pathway in patients with ESCC [69]. Wang et al*.* demonstrated that the expression level of *MIR4435-2HG* was upregulated in BCa tissues and cell lines compared to the control samples. They indicated that *MIR4435-2HG* served as a competing endogenous RNA (ceRNA) and sponged miR-4288. Using in vitro methods, they showed that knockdown of *MIR4435-2HG* significantly inhibited tumor growth by sponging miR-4288 [71]. Shu et al. reported that the high expression level of *MIR4435-2HG* was significantly associated with advanced tumor metastasis node in patients with HNSCC. In vivo and in vitro investigations indicated that knockdown of *MIR4435-2HG* decreased invasion, cell proliferation, and EMT in HNSCC cancer cell lines. As shown in Table 1, *MIR4435-2HG* executes these functions via modulating *miR‑383‑5p*. On the other hand, one target of *miR‑383‑5p* is *RNA binding motif protein 3 (RBM3)*. It can be concluded that HNSCC progression can be regulated by *MIR4435-2HG/miR‑383‑5p/RBM3* axis [70]. It has been established that flotillin-2 *(FLOT2)* has a critical role in the progression of human cancers through different mechanisms [74, 75]. According to bioinformatics analysis, *miR-802* targets *FLOT2* gene. Han et al*.* showed that *MIR4435-2HG* sponged *miR-802* which leads to increased expression of *FLOT2* and tumor progression (Table 1) [72]. The experimental studies showed that *MIR4435-2HG* inhibited apoptosis while facilitating migration, and cell proliferation in NPC cells. The mentioned lncRNA exerts this function by inhibiting of *phosphatase and tensin homolog* (*PTEN)* as a tumor suppressor gene [73].

## *MIR4435-2HG* and non-cancerous diseases

Accumulating evidence reveals that *MIR4435-2HG* not only is involved in cancer progression but also plays a critical role in the development of other diseases. In this section, we investigated the role of *MIR4435-2HG* in non-cancerous disorders.

### Coronary artery diseases (CAD)

The serum level of *MIR4435-2HG* remarkably increased in CAD patients compared to healthy controls. Clinical studies revealed that treatment with statins drugs, atorvastatin, and rosuvastatin, reduced *MIR4435-2HG* level significantly in CAD patients. This reduction was mainly observed in patients treated with rosuvastatin [12].

### Osteoarthritis

The *MIR4435-2HG* transcript level was lower in plasma samples of patients with osteoarthritis than in healthy controls. Knockdown of *MIR4435-2HG* decreased proliferation and promoted cell apoptosis in chondrocytes, while overexpression of *MIR4435-2HG* enhanced proliferation of chondrocytes and suppressed apoptosis. After treatment with anti-inflammatory drugs (such as naproxen), reducing the joint burden and exercise, the expression level of *MIR4435-2HG* was increased [14].

### Osteoporosis

Guang et al*.* reported that *MIR4435-2HG* was downregulated in plasma of patients suffering osteoporosis compared to healthy controls. They also showed a positive correlation between *MIR4435-2HG* and bone turnover markers, procollagen-1 N-terminal peptide (P1NP) and tartrate-resistant acid phosphatase 5b (TRACP-5b). The phenotype of osteoblasts can be regulated by type I collagen α1/α2 ratio. Knockdown of *MIR4435-2HG* suppressed α1 expression but upregulated α2. In contrast, upregulation of *MIR4435-2HG* elevated α1 but decreased α2. It can be concluded that *MIR4435-2HG* can affect the phenotype of osteoblasts via alteration in type I collagen α1/α2 ratio [15].

### Osteonecrosis of the femoral head (ONFH)

Runt‑related transcription factor 2 (*RUNX2*) has been identified as a marker of osteoblastic differentiation [76, 77]. Decreased expression of *RUNX2* led to the development of non‑traumatic ONFH [78]. The investigation showed that the expression level of *MIR4435-2HG* in both serum and mesenchymal stem cells (MSCs) samples was significantly downregulated. Silencing and overexpression of *MIR4435-2HG* in hMSC‑BM cells could lead to inhibition and promotion of *RUNX2* expression, respectively. To conclude, *MIR4435-2HG* participates in the progression of non‑traumatic ONFH through elevated *RUNX2* [13].

### Periodontitis

Xiaofang et al. demonstrated that the expression level of *MIR4435-2HG* was elevated in plasma samples of patients with periodontitis compared to healthy controls. They showed that the expression level of *MIR4435-2HG* was remarkably downregulated after treatment (administration of both oral and topical antibiotics, root planning and scaling). However, after two years of follow-up, the expression of *MIR4435-2HG* was significantly elevated in patients with recurrence of periodontitis [16].

### Human immunodeficiency virus (HIV) infection

Expression of *MIR4435-2HG* is also involved in immune responses against HIV-1 infection. Hartana et al. investigated the expression level of this lncRNA in myeloid dendritic cells (mDCs) obtained from HIV-1 elite controllers (ECs), in whom the virus replication is under control in the absence of antiretroviral treatment, compared to HIV-1-negative healthy controls and those who were treated using antiretroviral therapy. They found that *regulatory associated protein of MTOR complex 1 (RPTOR)* gene, a major component of the mammalian target of rapamycin (mTOR) signaling pathway, via induction of an epigenetic alteration. Taken together, upregulation of *MIR4435-2HG* in mDCs from ECs influences immunometabolic activities through different mechanisms, including altered glycolysis, oxidative phosphorylation, epigenetic modifications [79].

## Diagnostic value of *MIR4435-2HG*

Several studies have shown that evaluating lncRNAs expression in serum, plasma, and other body fluids may serve as diagnostic or prognostic biomarkers in different disorders that are non-invasive and convenient compared to biopsy and imaging methods. For example, Fu et al. showed the elevated levels of *MIR4435-2HG* both in tumor tissues and serum samples of gastric cancer patients. They suggested that this lncRNA may be a potential biomarker in gastric cancer [27]. Receiver Operating Characteristic (ROC) Curve Analysis plays a central role in evaluating the diagnostic ability of tests to discriminate the true state of subjects. The diagnostic value of *MIR4435-2HG* has been evaluated in some tumors and other diseases. *MIR4435-2HG* can differentiate tumor samples from corresponding controls and distinguish disease status in other non-cancerous conditions. As shown in Table 2, *MIR4435-2HG* has the best diagnostic power in osteoarthritis subjects.

**Table 2:** Diagnostic value of *MIR4435-2HG* in cancers and non-cancerous conditions [ALL: Acute lymphoblastic leukemia, AUC: Area under the Curve]

Disease	Expression	Number of samples	Sensitivity	Specificity	AUC	Sample	References
Gastric cancer	Up	51 cancer patients and 53 healthy controls	90.2	74.5	88.2	Plasma	[26]
Gastric cancer	Up	72 cancer patients and adjacent non-cancerous tissues	80	70	83.1	Serum	[27]
Hepatocellular cancer	Up	58 cancer patients and 45 healthy controls	75.9	95.9	91	Serum	[36]
Colorectal cancer	Up	70 cancer patients and adjacent non-cancerous tissues	72	80	81	Tissue	[21]
Colon cancer	Up	46 cancer patients and 42 healthy controls	–	–	84.8	Serum	[20]
Ovarian carcinoma	Up	66 cancer patients and 54 healthy controls	–	–	88.2	Plasma	[50]
ALL	Up	32 cancer patients and 32 healthy controls	–	–	89.5	Bone marrow	[64]
Renal cell carcinoma	Up	118 cancer patients and adjacent non-cancerous tissues	–	–	94.6	Tissue	[67]
Osteoarthritis	Down	78 osteoarthritis and 58 healthy controls	–	–	96	joint fluid	[14]
Osteoporosis	Down	88 osteoporosis patients and 57 healthy control	–	–	92	plasma	[15]
Non-traumatic ONFH	Down	36 ONFH patients and 30 healthy controls	–	–	81.8	Serum	[13]

## Conclusion

*MIR4435-2HG* participates in the progression of different human disorders. *MIR4435-2HG* exerts its functions via the spectrum of different mechanisms, including inhibition of apoptosis, sponging miRNAs, promotion of cell proliferation, increasing cell invasion and migration, and enhancement of EMT. As mentioned above, different miRNAs such as *miR-6754-5p*, *miR-1224-5p*, *miR-802*, and *miR-128-3p* can be sponged by *MIR4435-2HG*. On the other hand, *MIR4435-2HG* can lead to tumor progression by affecting Wnt, TGF-β/SMAD, Nrf2/HO-1, PI3K/AKT, MAPK/ERK, and FAK/AKT/β‑catenin signaling pathways. Several studies have shown that *MIR4435-2HG* acts as an oncogene in different types of cancer.

The overexpression of *MIR4435-2HG* in all cancer types that have been studied so far indicates the key role of this lncRNA in cancer progression as an oncogene. Cell proliferation, EMT, invasion, migration, and suppressed apoptosis are key hallmarks of cancer that can be affected by *MIR4435-2HG* expression. Besides, several studies confirmed the effectiveness of *MIR4435-2HG* silencing in inhibiting tumor growth in colorectal cancer, esophageal squamous cell carcinoma, gastric cancer, hepatocellular carcinoma, lung cancer, neuroglioma, and prostate cancer.

In contrast, in non-cancerous conditions such as periodontitis, osteoporosis, osteoarthritis, and osteonecrosis of the femoral head, the expression level of *MIR4435-2HG* has been downregulated. However, in coronary artery diseases, the expression level of *MIR4435-2HG* was elevated.

The expression level of *MIR4435-2HG* alters in response to many drugs, including statins (atorvastatin and rosuvastatin), oral and topical antibiotics, anti-inflammatory drugs (such as naproxen) and chemopreventive agent resveratrol. This subject indicates that *MIR4435-2HG* has a pivotal function in molecular mechanisms involved in disease development. Therefore, it can be concluded that *MIR4435-2HG* may serve as a potential therapeutic target for the treatment of various diseases.

Despite fundamental improvement in cancer diagnosis methods, recurrence and metastasis occur in many patients suffering from cancer, therefore, the discovery of new diagnostic biomarkers could be helpful in this regard [80, 81]. Moreover, according to the literature, the diagnostic value of *MIR4435-2HG* was acceptable in both cancerous and non-cancerous conditions. Detection and measurement of *MIR4435-2HG* in body fluids such as serum, plasma, and joint fluid suggest that this lncRNA could be used as a non-invasive marker.

Although previous studies have emphasized the role of *MIR4435-2HG* in the progression of different diseases, few studies has been conducted to describe the possible mechanisms involved in its regulation. Therefore, understanding the mechanisms involved in *MIR4435-2HG* regulation may shed light on the diagnosis and treatment of several related diseases.

In conclusion, *MIR4435-2HG* has a pivotal role in cancer progression and critical function in non-neoplastic conditions. Future studies may explain the role of this lncRNA as a potential biomarker and therapeutic target in human disorders, especially in tumors.

## Data Availability

Not applicable.

## Abbreviations

lncRNA: Long non-coding RNA
miRNAs: MicroRNAs
EMT: Epithelial-mesenchymal transition
*MIR4435-2HG*: *MIR4435-2 Host Gene*
YAP1: Yes-related protein 1
Nrf2: Nuclear factor erythroid 2-related factor 2
HO-1: Hemeoxygenase-1
*GLUT‑1*: *Glucose transporter 1*
p-SMAD2: Phosphorylated SMAD2
NTRK3: Tropomyosin rseceptor kinase C
*SOX4*: *SRY-box transcription factor 4*
YBX1: Y-box binding protein 1
SNAIL1: Snail family transcriptional repressor 1
PIK3CA: Phosphatidylinositol-4,5-bisphosphate 3-kinase catalytic subunit alpha
PRC2: Polycomb repressive complex
EZH2: Enhancer of zeste homolog
ChIP: Chromatin immunoprecipitation
p-JNK: Phosphorylated JNK
p-ERK: Phospho-ERK
MAPK: Mitogen-activated protein kinase 1
*B3GNT5*: *UDP-GlcNAc:betaGal beta-1,3-N-acetylglucosaminyltransferase 5*
*CDK6*: *Cyclin dependent kinase 6*
TGF- β1: Transforming growth factor-β1
*CDK14*: *Cyclin dependent kinase 14*
FAK: Focal adhesion kinase
*ST8SIA1*: *ST8 alpha-N-acetyl-neuraminide alpha-2,8-sialyltransferase 1*
mTOR: Mammalian target of rapamycin
TGFBR2: Transforming growth factor-beta receptor type II
TAZ: Tafazzin, phospholipid-lysophospholipid transacylase
ROCK2: Rho‑associated protein kinase 2
*MSI2*: *Musashi RNA binding protein 2*
*KLF6*: *Kruppel like factor 6*
ceRNA: Competing endogenous RNA
*RBM3*: *RNA binding motif protein 3*
*PTEN*: *Phosphatase and tensing homology*
P1NP: Procollagen-1 N-terminal peptide
TRACP-5b: Tartrate-resistant acid phosphatase 5b
RUNX2: Runt‑related transcription factor 2
MSDs: Mesenchymal stem cells
HMSC-bm: Human Mesenchymal Stem Cells-Bone Marrow
*RPTOR*: *Regulatory associated protein of MTOR complex 1*
ROC: Receiver operating characteristic
CRC: Colorectal cancer
GC: Gastric cancer
HCC: Hepatocellular carcinoma
NSCLC: Non‑small cell lung cancer
OC: Ovarian cancer
PC: Prostate cancer
GBM: Glioblastoma
T‑ALL: T‑cell acute lymphoblastic leukemia
AML: Acute myeloid leukemia
CC: Cervical cancer
ccRCC: Clear cell renal cell carcinoma
ESCC: Esophageal squamous cell
HNSCC: Head and neck squamous cell carcinoma
BCa: Bladder cancer
NPC: Nasopharyngeal carcinoma
BC: Breast cancer
LC: Lung cancer
